# Distal, intermediate, and proximal mediators of racial disparities in renal disease mortality in the United States

**DOI:** 10.15171/jnp.2016.09

**Published:** 2015-12-04

**Authors:** Shervin Assari

**Affiliations:** ^1^Department of Psychiatry, University of Michigan, Ann Arbor, MI, USA; ^2^Center for Research on Ethnicity, Culture and Health, School of Public Health, University of Michigan, Ann Arbor, MI, USA

**Keywords:** Ethnic groups, African Americans, Socioeconomic status, Hypertension, Diabetes, Obesity, Mortality, Renal diseases

## Abstract

*Background:* Kidney failure and associated mortality is one of the major components of racial disparities in the United States.

*Objectives:* The current study aimed to investigate the role of distal (socioeconomic status, SES), intermediate (chronic medical diseases), and proximal (health behaviors) factors that may explain Black-White disparities in mortality due to renal diseases.

*Patients and Methods:* This is a nationally representative prospective cohort with 25 years of follow up. Data came from the Americans’ Changing Lives (ACL) study, 1986 to 2011. The study included 3361 Black (n = 1156) or White (n = 2205) adults who were followed for up to 25 years. Race was the main predictor and death due to renal disease was the outcome. SES, chronic medical disease (diabetes, hypertension, obesity), and health behaviors (smoking, drinking, and exercise) at baseline were potential mediators. We used Cox proportional hazards models for data analysis.

*Results:* In age and gender adjusted models, Blacks had higher risk of death due to renal disease over the follow up period. Separate models suggested that SES, health behaviors and chronic medical disease fully explained the effect of race on renal disease mortality.

*Conclusions:* Black-White disparities in rate of death due to renal diseases in the United States are not genuine but secondary to racial differences in income, health behaviors, hypertension, and diabetes. As distal, intermediate, and proximal factors contribute to racial disparities in renal disease mortality, elimination of such disparities requires a wide range of policies and programs that target income, medical conditions, and health behaviors.

**Implication for health policy/practice/research/medical education:**

In the United States, racial disparities in mortality due to renal disease are due to distal (socioeconomics), intermediate (hypertension and diabetes), and proximal (health behaviors) factors. Thus, elimination of racial disparities in renal disease mortality in this country requires a wide range of policies and programs that enhance income, promote behaviors, and prevent medical conditions such as hypertension and diabetes.

## 1. Background

In the United States, Blacks are at higher risk of mortality due to renal diseases (1,2). This is mainly because compared to Whites, Blacks are 3-4 times more likely to develop kidney failure. While Blacks only make up to 13% of the population, they account for one third of all kidney failures in the United States (1). It is still unknown whether or not racial disparities in renal disease mortality are genuine (i.e. due to biological factors such as genetic predisposition) or they are secondary to Black-White differences in 1) socioeconomic status (SES), 2) chronic medical disease (e.g. hypertension, diabetes, and obesity), or 3) health behaviors (e.g. exercise, smoking, and drinking).

First, SES may be distal mediator of the link between race and burden of kidney disease. In the United States, race is a proxy of social class, and a considerable proportion of racial differences in health is in fact due to class, education, income, or wealth (3-6). Compared to Whites, a lower number of Blacks receive high quality education (7). Blacks are at higher risk of school dropout than Whites (8). Even among highly educated individuals, a large gap exists in income and wealth of Blacks compared to Whites, which is mostly due to structural racism and societal factors (9-12).

Second, chronic medical diseases (e.g. hypertension, diabetes and obesity) may also be intermediate mediators of the association between race and renal disease mortality. Hypertension and diabetes are leading causes of kidney failure among Blacks (1). As a result, at least some of the racial disparities in burden of renal disease may be secondary to higher prevalence of chronic medical conditions among Blacks (13,14). Hypertension (15), diabetes (16), and obesity (17) which are known causes of kidney disease are all more common among Blacks (18-21). The role of diabetes (22,23) and hypertension (24,25) as etiologic factors in development of chronic kidney disease are well known. Obesity, which is more common among Blacks compared to Whites, also increases the risk of chronic renal disease (26).

Finally, health behaviors such as exercise, smoking, and drinking may be proximal explanatory factors (i.e. mediators) that explain at least some of the racial disparities in renal disease mortality. We know that the distribution of health behaviors such as physical activity, smoking, and drinking vary across population groups (27). Exercise (28), smoking (29), and drinking (30) all differ between Whites and Blacks. All of these behaviors influence development and progression of renal diseases (31-34).

For at least three reasons, more studies are needed to explore whether or not racial disparities in renal disease mortality are genuine or secondary to racial gaps in SES, chronic medical disease, or health behaviors. First, very few studies have focused on mediators of racial differences in renal disease mortality (2). Second, although some studies have shown that fatality of renal disease depends on race (26,35-38), we still do not know the mechanisms behind such disparities. Third, most prior research has used local samples, and most studies with a prospective design have used a short-term follow up period. Thus, more studies are needed which follow a nationally representative sample for a long-term period.

## 2. Objectives

To better understand mechanisms behind racial disparities in burden of renal disease in the United States, we examined roles of distal (SES), intermediate (chronic medical disease), and proximal (health behaviors) factors that may explain racial inequalities in mortality due to renal diseases between Blacks and Whites.

## 3. Patients and Methods

### 3.1. Study design

### 3.1.1. Setting

Data came from the Americans’ Changing Lives (ACL), a nationally-representative U.S. cohort study conducted from 1986 until 2011. Detailed information on the study design is available elsewhere (39,40).

### 3.2.2. Sampling and participants

The ACL enrolled a stratified multistage probability sample of adults ages 25 or above who lived in the continental U.S. in 1986. The study included 3617 non-institutionalized respondents (representing 70% of sampled households and 68% of sample individuals at baseline) with an oversampling of those age 60 and older, and African Americans. Wave 1 included 70% of sampled households and 68% of sampled individuals. Further interviews were conducted in 1989, 1994, 2001-2002 and 2011, but information from those interviews was not relevant for these analyses.

### 3.2.3. Measures

Information on demographics, SES, chronic medical conditions, and health behaviors was measured at the baseline during face to face interview in 1986.

### 3.2.4. Race

The predictor was race defined as non-Hispanic Black or non-Hispanic White based on a coding of self-reported items asking about Hispanic ethnicity, nativity, and racial category.

### 3.3. Demographic factors

Demographic indicators included age (a continuous variable as number of years since birth) and gender (a dichotomous variable with male as the referent category).

### 3.4. Socioeconomic characteristics

Data on SES were measured with an indicator of education (less than 12 years of education, and 12 years or more) and income (10 level categorical variable treated as a continuous measure; <$5000, $5-9K, $10-14K, $15-19K, $20-24K, $25-29K, 30-39K, $40-59K, $60-79K, $80 000+).

### 3.4.1. Diabetes and hypertension

Self-reported history of hypertension and diabetes were assessed at baseline. Using separate items, all participants were asked whether a health care provider had ever told them they had hypertension or diabetes (40,41).

### 3.4.2. Obesity

The body mass index (BMI) was calculated based on self-reported weights and heights. Weight and height were originally collected in pounds (1 pound = 0.453 kg) and feet (1 foot = 0.3048 m)/inches (1 inch = 0.0254 m), respectively. Obesity was defined as BMI equal to or larger than 30 kg/m^2^ (42). BMI calculated based on self-reported weight and height is known to be closely correlated with BMI based on direct measures of height and weight (43). However, using self-reported weight and height may lead to some degrees of underestimation of BMI (44), because of a systematic tendency for humans to underestimate their weight and to overestimate their height (45).

### 3.5. Health behaviors

We collected data on self-reported smoking (current smoker vs. other), drinking (current drinker vs. other), and exercise (frequency of physical activities) using the following single-item measures. Do you smoke cigarettes now? Do you ever drink beer, wine, or liquor? How often do you engage in active sports or exercise — would you say often, sometimes, rarely or never? Responses of the first two items were yes and no, and responses of the third item were 1) often, 2) sometimes, 3) rarely, and 4) never.

### 3.6. Mortality due to renal diseases

The main outcome variable was time of death due to renal diseases. Information on all deaths from mid-1986 through 2011 was obtained through the National Death Index (NDI), death certificates, and also from informants. In most cases, time and cause of death were verified with death certificates. The handful of cases where death could not be verified with death certificates were reviewed carefully, and actual death was certain in all cases. Only in these cases, was the date of death ascertained from the informants or the NDI report, rather than the death certificate (41,46). Cause of death was coded as unknown if death certificate or NDI report were unavailable.

We used the ICD-9 and ICD-10 codes (47,48), whichever was current at the time the death was recorded, to determine death due to renal diseases (kidney-urinary). For ICD-9 codes, we used codes 650 (acute glomerulonephritis and nephrotic syndrome), 660 (chronic glomerulonephritis, nephritis, and nephropathy, not specified as acute or chronic, and renal sclerosis, unspecified), 670 (renal failure, disorders resulting from impaired renal function, and small kidney of unknown causes), 680 (infections of kidney), and 690 (hyperplasia of prostate). For ICD-10 codes, we used the categorization of 113 selected causes of death provided by World Health Organization (WHO), for which codes 97 (nephritis, nephrotic syndrome, and nephrosis), 98 (acute and rapidly progressive nephritic and nephrotic syndrome), 99 (chronic glomerulonephritis, nephritis, and nephropathy not specified as acute or chronic, and renal sclerosis unspecified), 100 (renal failure), 101 (other disorders of kidney), 102 (infections of kidney), 103 (hyperplasia of prostate), and 104 (inflammatory diseases of female pelvic organ) were used. Respondents who died due to other causes were censored at the time of death. Time of death was registered as number of months from time of enrollment to the study to time of death, based on the month of death and the month of the baseline interview.

### 3.7. Ethical issues

The research followed the tenets of the Declaration of Helsinki. This project was approved by the institutional review board (IRB) of the University of Michigan, Ann Arbor. All participants provided written consent and all data were kept confidential.

### 3.8. Statistical analysis

Univariate, bivariate, and multivariable analyses were performed using Stata 13.0 (Stata Corporation, College Station, TX, USA). Stratification and clustering in the estimation of standard errors was accounted for by using Taylor series linearization. *P* < 0.05 was considered statistically significant. Adjusted hazard ratios (HR) with 95% CI are reported.

For multivariable analysis, six Cox proportional hazards models were fitted to the data. Cox proportional hazards models require a binary outcome (renal death) and time to the event or to censoring, defined as the number of months from baseline to death, loss to follow up, or the end of the year 2011. Renal death was coded zero if the respondent did not die, or died from any other causes. Race was the main predictor. Baseline SES (education and income), health behaviors (smoking, drinking and exercise) and chronic medical disease (hypertension, diabetes, and obesity) were potential mediators.

## 4. Results

Table 1 shows descriptive statistics for the overall sample, and separately for Whites and Blacks. Whites and Blacks did not differ in age or gender. However, compared to Whites, Blacks had lower education and income, smoked more, drank less, and had more hypertension (HTN), diabetes mellitus (DM) and obesity (All differences were significant at *P* < 0.05). Death due to renal disease was also more common among Blacks than Whites.

**Table 1:** Descriptive statistics overall and based on race at baseline

	** All**		**Whites**		**Blacks**	
	**Mean (SE)**	**95% CI**	**Mean (SE)**	**95% CI**	**Mean (SE)**	**95% CI**
Age	47.79 (0.53)	46.72-48.86	47.98 (0.60)	46.77-49.19	46.37 (0.71)	44.93-47.81
Income^a^	5.41 (0.09)	5.22-5.60	5.57 (0.10)	5.36-5.77	4.25 (0.18)	3.88-4.62
Exercise^a^	0.02 (0.03)	-0.03-0.07	0.06 (0.03)	0.00-0.11	-0.22 (0.05)	-0.33-0.12
Education^a^	12.53 (0.10)	12.34-12.73	12.69 (0.11)	12.48-12.90	11.37 (0.23)	10.90-11.84
	**% (SE)**	**95 % CI**	**% (SE)**	**95 % CI**	**% (SE)**	**95 % CI**
Gender						
Male	47.26 (0.01)	44.86-49.68	47.82 (0.01)	45.12-50.52	43.18 (0.02)	38.79-47.69
Female	52.74 (0.01)	50.32-55.14	52.18 (0.01)	49.48-54.88	56.82 (0.02)	52.31-61.21
HTN^a^	21.37 (0.01)	19.67-23.17	19.77 (0.01)	18.11-21.53	33.17 (0.03)	28.20-38.54
DM^a^	5.73 (0.00)	4.80-6.82	5.25 (0.01)	4.24-6.50	9.22 (0.01)	7.75-10.95
Obesity^a^	14.47 (0.01)	12.86-16.24	13.52 (0.01)	11.72-15.54	21.45 (0.02)	17.88-25.52
Smoking^a^	30.45 (0.01)	27.81-33.23	29.70 (0.01)	26.85-32.72	35.98 (0.03)	30.81-41.49
Drinking^a^	60.02 (0.02)	56.68-63.26	61.50 (0.02)	58.06-64.83	49.10 (0.03)	43.55-54.68
Renal death	0.52 (0.00)	0.26-1.03	0.44 (0.00)	0.18-1.06	1.15 (0.00)	0.63-02.09

Abbreviations‏: CES‏-D‏, Center‏ for‏ Epidemiologic‏ Studies‏ Depression‏ Scale‏; HTN‏, hypertension‏; DM‏, diabetes‏ mellitus‏; SE, standard error‏. ^a^ *P* < 0.05.

Table 2 shows a summary of the results of six Cox proportional hazard regression models. According to the results of Model 1, which only adjusted for age and gender, Blacks were at higher risk of death due to renal disease. The adjusted hazard ratio for race did not remain significant in any Models 2 to 6 which controlled for SES, health behaviors, and chronic medical conditions.

**Table 2:** Summary of Cox regressions on mediators of the association between race and death due to renal disease in the United States

	**Model 1** **Race + Demographics**	**Model 2** **M1 + SES**	**Model 3** **M1 + CMCs**	**Model 4** **M1 + CMC + Obesity**	**Model 5** **M1 + Health Behaviors**	**Model 6** **Full**
	**HR (95% CI)**	**HR (95% CI)**	**HR (95% CI)**	**HR (95% CI)**	**HR (95% CI)**	**HR (95% CI)**
Black	3.31^a^(1.15-9.49)	2.32^d^(0.96-5.58)	2.00 (0.61-6.59)	1.98 (0.64-6.07)	2.26 (0.69-7.41)	1.87 (0.87-4.01)
Age	1.09^c^(1.06-1.12)	1.08^c^(1.06-1.10)	1.07^b^(1.01-1.12)	1.07^b^(1.02-1.12)	1.09^c^(1.06-1.11)	1.07^c^(1.05-1.10)
Female	0.65 (0.16-2.61)	0.49 (0.12-2.00)	0.65 (0.17-2.47)	0.60 (0.15-2.49)	0.40 (0.08-1.93)	0.30 (0.07-1.30)
Education (y)	-	1.00 (0.86-1.17)	-	-	-	1.08 (0.92-1.26)
Income	-	0.76^b^(0.64-0.90)	-	-	-	0.80^d^(0.63-1.01)
DM	-	-	8.98^b^(2.41-33.44)	7.65^c^(2.48-23.57)	-	6.58^c^(2.76-15.67)
HTN	-	-	4.29^a^(1.30-14.10)	3.61^a^(1.36-9.62)	-	2.90^a^(1.18-7.09)
Obesity	-	-	-	2.14 (0.70-6.53)	-	1.72 (0.60-4.95)
Smoking	-	-	-		3.43^a^(1.07-11.01)	3.59^a^(1.26-10.24)
Drinking	-	-	-		0.10^b^(0.02-0.46)	0.14^b^(0.04-0.47)
Exercise	-	-	-		0.67^d^(0.43-1.04)	0.72 (0.43-1.19)

Abbreviations: SES, socioeconomic status; HTN, hypertension; DM, diabetes mellitus; M1, Model 1; HR, hazard ratio‏. ^a^‏ *P*‏ < 0.05; ^b^*P*‏ < 0.01; ^c^*P*‏ < 0.001; ^d^*P*‏ < 0.1

## 5. Discussion

According to our findings, in age and gender adjusted models, race was associated with death due to renal disease over the follow up period. In separate models, SES (income), health behaviors (smoking, drinking, and exercise) and chronic medical disease (diabetes and HTN) fully explained the effect of race on death due to renal disease.

First, this study provided support for SES as a distal mediator of racial disparities in rate of death due to renal disease. It has previously shown that SES is a major determinant of behaviors and health outcomes (49-51). According to the fundamental cause theory (FCT), developed by Link and Phelan, low SES is a root cause of health problems including high-risk behaviors (52-55). Link and Phelan have listed four essential features for SES as fundamental causes of health inequalities (55). First, the effect of SES is not limited to specific health problems as it influences most health outcomes. Second, SES affects health through multiple risk factors and mechanisms. Third, SES involves access to resources that can be used to avoid health risks or to minimize the consequences of health problems once they occur. Finally, SES is continually influencing health inequalities despite radical changes in causes of morbidity and mortality over the past several decades (55).

Second, we found that HTN and diabetes are two intermediate mediators of such disparities. Diabetes and high blood pressure are leading causes of kidney failure among African Americans (1). As a result, at least some of the racial disparity in rate of death due to renal disease may be due to chronic medical conditions (13,14) such as HTN (15), diabetes (16), and obesity (17) which are more common among Blacks (18-21). A well-established literature shows that diabetes is associated with chronic kidney disease (22). In fact chronic kidney disease is one of the complications of diabetes (23). The association between chronic kidney disease and HTN is also bidirectional, and HTN is one of the known etiologic factors in development of chronic kidney disease (25). Longitudinal studies have shown that end stage renal disease is one of many potential outcomes in patients with HTN (18).

Third, health behaviors such as exercise, smoking, and drinking seem to be the most proximal mediators that explain at least some of the racial disparities in rate of death secondary to renal diseases. Distribution of exercise (28), smoking (29), and drinking (30) all differ between Whites and Blacks and all impact development and progression of renal diseases (31-34). While we know that the distribution of health behaviors vary across population groups (26,27), we know less about effective ways to reduce such disparities. Research has shown that culturally sensitive tailored interventions may have better effects in promoting health of minority groups including Blacks (56,57).

Our findings suggest that racial disparities in rate of death due to renal disease are secondary to racial gaps in SES (income), chronic medical disease (diabetes and HTN), and health behaviors (smoking, drinking, and exercise). Our findings are against researchers who believe that differences in the burden of renal disease are genuine (22) and due to a higher disposition of Blacks having renal disease due to biological factors such as genetics. Although we cannot rule out the role of biological factors, we believe there is a need to study root causes of such disparities inside the United States instead of conducting studies on biological causal paths of hypertensive disease, diabetes, and renal risk in Black Africa (58). We argue that psychosocial disparities not genetic factors are causing racial disparities in death due to renal disease in the United States. Thus our finding is against the belief that higher susceptibility of Blacks to these diseases is through the direct genetic propensity of Blacks (19,20).

In line with other studies, we believe that it is not race per se, that causes disparities in diabetes, HTN, or renal disease, (25) but such disparities are the result of a wide range of social, medical, and behavioral factors. Distribution of other risk factors also differs between Whites and Blacks (19). Similar findings in other chronic conditions have suggested that not race, per se, but SES and other risk factors explain racial differences in outcomes (59-61).

Multiple studies have decomposed the effects of race, SES, and risk factors on health. Our study is, however, different from those who measure additive effects of race, SES, and medical conditions on outcomes (62-64). For instance, a number of studies have shown that race has a residual main effect above and beyond SES and other confounders on self-care and knowledge in HTN, diabetes, and other conditions (63,64). The real association between race, SES, and health is, however, complex and multiplicative rather than additive (65,66). The same risk factors including SES may not have the same effects across race groups (67). Some of these studies have shown that Blacks and Whites would show similar outcomes if they could have similar SES (68). Other studies have shown that SES interacts with race on outcomes (69). Farmer and Ferraro, for instance, showed that racial disparity in self-rated health was largest at the higher levels of SES, providing some evidence for the “diminishing returns” hypothesis. As education levels increases, Blacks may not have the same improvement in health as Whites (69).

In a study, Perneger et al conducted a case-control study to compared 716 patients with end-stage renal disease (ESRD) with 361 population controls. Race, indicators of SES, and indicators of access to health care were assessed. Authors showed that adjustment for SES partially explained the odds ratio for Blacks. The proportions of ESRD incidence that could be attributed to race and income were 46% and 53% respectively. Authors showed that limited access to health care also explains some of the excess of ESRD in Blacks (70). Other studies have explored the role of the health care system or relation between patient and system as possible mediators of such disparities (71).

## 6. Conclusions

To conclude, we found that Black-White differences in rate of death due to renal diseases over a 25-year period are due to a complex network of distal, intermediate, and proximal factors, namely SES (income), chronic medical disease (diabetes and HTN), and health behaviors (smoking, drinking, and exercise), respectively. Our findings extend the existing knowledge on racial disparities in renal disease and associated mortality. This is particularly important as Black-White disparity in chronic kidney disease is a major challenge in the United States (72).

## 7. Limitations of the study

Despite the unique contribution that the current study makes to the literature, the results should be interpreted with consideration of the study limitations. The first limitation was lack of any measure of kidney disease at baseline or over course of the follow up. In addition, measurement of chronic medical conditions (HTN, diabetes, and obesity) was based on self-reported data, which is subject to recall bias (73). Further research can use multiple sources regarding the history of medical conditions. Future studies should assess how race, SES, behaviors, and clinically-diagnosed HTN and diabetes explain disparities in decline in kidney function and developments of end stage renal disease among Blacks and Whites. Despite these limitations, the study had major strengths, including a long term follow up, a nationally representative U.S. sample and a large sample of Blacks.

## Author’s contribution

SA is the single author of the manuscript. SA designed the study, analyzed the data, drafted the paper, and revised the manuscript.

## Conflicts of interest

SA declares that he has no conflicts of interest.

## Funding/Support

The Americans’ Changing Lives (ACL) study was supported by Grant # AG018418 from the National Institute on Aging (DHHS/NIH), and per the NIH Public Access Policy requires that peer-reviewed research publications generated with NIH support are made available to the public through PubMed Central. NIH is not responsible for the data collection or analyses represented in this article. The ACL study was conducted by the Institute of Social Research, University of Michigan. Shervin Assari is supported by the Heinz C. Prechter Bipolar Research Fund and the Richard Tam Foundation at the University of Michigan Depression Center.
